# Impact of socio-economic factors on Tuberculosis treatment outcomes in north-eastern Uganda: a mixed methods study

**DOI:** 10.1186/s12889-021-12056-1

**Published:** 2021-11-26

**Authors:** Jasper Nidoi, Winters Muttamba, Simon Walusimbi, Joseph F. Imoko, Peter Lochoro, Jerry Ictho, Levicatus Mugenyi, Rogers Sekibira, Stavia Turyahabwe, Raymond Byaruhanga, Giovanni Putoto, Simone Villa, Mario C. Raviglione, Bruce Kirenga

**Affiliations:** 1 Makerere University Lung Institute (MLI), Kampala, Uganda; 2 Doctors with Africa CUAMM, Kampala, Uganda; 3 National Tuberculosis and Leprosy Control Program (NTLP), Kampala, Uganda; 4 Doctors with Africa CUAMM, Padova, Italy; 5 Centre for Multidisciplinary Research in Health Science, University of Milan, Milan, Italy

**Keywords:** Tuberculosis treatment outcomes, Socio-economic factors, Determinants of health

## Abstract

**Background:**

Tuberculosis (TB) is a major public health problem and at 48%, Karamoja in North-Eastern Uganda has the lowest treatment success rate nationally. Addressing the social determinants of TB is crucial to ending TB. This study sought to understand the extent and ways in which socio-economic factors affect TB treatment outcomes in Karamoja.

**Methods:**

We conducted a convergent parallel mixed methods study in 10 TB Diagnostic and Treatment Units. The study enrolled former TB patients diagnosed with drug-susceptible TB between April 2018 and March 2019. Unit TB and laboratory registers were reviewed to identify pre-treatment losses to follow-up. Four focus group discussions with former TB patients and 18 key informant interviews with healthcare workers were conducted. Principle component analysis was used to generate wealth quintiles that were compared to treatment outcomes using the proportion test. The association between sociodemographic characteristics and TB treatment outcomes was evaluated using the chi-square test and multiple logistic regression.

**Results:**

A total of 313 participants were randomly selected from 1184 former TB patients recorded in the unit TB registers. Of these, 264 were contacted in the community and consented to join the study: 57% were male and 156 (59.1%) participants had unsuccessful treatment outcomes. The wealthiest quintile had a 58% reduction in the risk of having an unsuccessful treatment outcome (adj OR = 0.42, 95% CI 0.18–0.99, *p* = 0.047). People who were employed in the informal sector (adj OR = 4.71, 95% CI 1.18–18.89, *p* = 0.029) and children under the age of 15 years who were not in school or employed (adj OR = 2.71, 95% CI 1.11–6.62, p = 0.029) had significantly higher odds of unsuccessful treatment outcome. Analysis of the pre-treatment loss to follow-up showed that 17.2% of patients with pulmonary bacteriologically confirmed TB did not initiate treatment with a higher proportion among females (21.7%) than males (13.5%). Inadequate food, belonging to migratory communities, stigma, lack of social protection, drug stock-outs and transport challenges affected TB treatment outcomes.

**Conclusions:**

This study confirmed that low socio-economic status is associated with poor TB treatment outcomes emphasizing the need for multi- and cross-sectoral approaches and socio-economic enablers to optimise TB care.

**Supplementary Information:**

The online version contains supplementary material available at 10.1186/s12889-021-12056-1.

## Background

Globally, tuberculosis (TB) comes second only to COVID − 19 as a leading cause of mortality from a single infectious organism, accounting for 1.4 million deaths annually [1, 2]. The epidemiological impact of available therapeutic and preventive strategies on pertinent TB indicators has been slow, resulting in the growing recognition of the role of social determinants of health at an individual and societal level on strategies to end TB as a public health problem [3–6].

The End TB Strategy and the United Nations Sustainable Development Goals (SDGs) recognize the interdependence between social determinants and health [4, 7–9]. The conditions in which people are born, grow, live, work and age act on a population, resulting in health disparities which are particularly important in TB where striking inequalities are well recognised [10]. Globally, over 90% of TB patients are in low- and middle-income countries and the cases remain mainly clustered among economically and socially disadvantaged groups [6, 11, 12]. Social status and daily living conditions modify several risk factors over time and influence access to resources leading to differential exposure, differential vulnerability to disease-causing and/or modifying agents and differential consequences of ill health [13, 14].

An association between social determinants of health and treatment outcomes has been established. Patients of low socio-economic status (SES) are less likely to seek medical help, get appropriate investigations for TB, have good TB treatment outcomes and they incur catastrophic costs [15, 16]. Predictors of poor treatment outcomes associated with socio-economic deprivation include low income, low education, high alcohol intake, long travel times, rural residence and under-nutrition [17–22]. A review of three socio-economic determinants found that not only were low income and alcohol abuse significantly associated with treatment failure but also increased the risk of developing multi-drug resistant TB; however, low education levels were only associated with treatment failure [23]. HIV is known to increase the vulnerability to TB and it has been associated with poor TB treatment outcomes [24, 25] particularly among patients not on anti-retroviral therapy [25–28]. The healthcare system is another important social determinant of health and a study in South Africa demonstrated that poorer regions had higher rates of drug stock-outs and in turn, drug stock-outs significantly reduced TB treatment cure and success rates [29]. In Mozambique, a lack of laboratory confirmation of TB probably due to misdiagnosis or paucibacillary TB among immune-suppressed people living with HIV was significantly associated with higher mortality [28].

Addressing the social determinants of health is pivotal in ending TB. A modelling study on the impact of ending extreme poverty and expanding social protection coverage under SGD 1 found that their combined direct effects would result in an 84∙3% reduction in TB incidence [30]. Indeed, conditional cash transfers [31] and economic enablers [32] given during TB treatment have been found to improve treatment outcomes, possibly through a reduction in loss to follow-up (LTFU) rates [32, 33]. In real-world settings, poor implementation of economic enabler programmes may attenuate the desired effects on TB treatment outcomes. A trial done in South Africa showed non-significant improvements in treatment outcomes from economic enablers because of low fidelity to the intervention with over a third of eligible patients not receiving the intervention [34].

Uganda is listed among the 30 high TB/HIV burden countries with a national TB prevalence of 253/100,000, HIV prevalence among those aged 15–64 of 6.2% and a 41% HIV coinfection rate among notified TB patients [35, 36]. Karamoja, a region located in the North-East, has the highest crude TB prevalence (2394/100,000 population), highest case notification rate (230/100,000 population) and lowest treatment success rate (48%) [36]. This region also has the highest proportion of persons that are poor [37, 38]. In the populations, diverse social-cultural practices aided by high levels (74.2%) of poverty and low (33%) literacy also contribute negatively to health service utilisation and outcomes.

TB patients initiated on treatment who fail to achieve desired outcomes are at risk of death, developing drug-resistant TB, and perpetuating TB transmission in their community. This study sought to document the magnitude and ways in which socio-economic factors affect TB treatment outcomes and the proportion of pre-treatment LTFU among TB patients. The study employed the social causation hypothesis which alludes to low social status as a precursor for ill health [39, 40]. We hypothesized that economically and socially disadvantaged groups lack financial, nutritional and social support and will ultimately encounter challenges in completing TB treatment resulting in poor outcomes. The framework for proximate risk factors and upstream determinants of TB [6] was used to identify and analyse social determinants of health and their association to TB outcomes.

## Methods

### Study design

We conducted a convergent parallel mixed methods [41] study in which former TB patients diagnosed with drug-susceptible TB and entered in the unit TB registers between April 2018 and March 2019 were enrolled into a retrospective cohort. Focus group discussions (FGDs) with former TB patients and key informant interviews (KII) with healthcare personnel involved in TB care were conducted to capture patient experiences and health system factors that could affect TB treatment outcomes. This study aimed to assess the relationship between social determinants of health and TB treatment outcomes in the Karamoja region. Mixed methods were selected to extend the breadth of inquiry on the impact of socio-economic factors on TB treatment outcomes: quantitative methods were used to establish the magnitude of the association between socio-economic factors and TB treatment outcomes while FGDs and KIIs were used to identify perceptions on how conditions of daily life and healthcare system factors acted as barriers or motivators during TB treatment. Data was collected simultaneously between 10th February and 24th March 2020.

### Study setting

The study was conducted in Karamoja, a rural economically deprived region in North-Eastern Uganda [37, 38]. Data were collected in 10 TB Diagnostic & Treatment Units (DTUs) in the five southern districts of Moroto, Napak, Amudat, Nabilatuk and Nakapiripirit. Electronic data from the District Health Information System [42], which includes records of notified TB cases, was analysed to identify DTUs for the study. The system is hosted by the Ministry of Health, Uganda for reporting routine Health Management Information System data. For feasibility purposes, the 10 DTUs selected had the highest case notification volume between April 2018 and March 2019 and managed 74.8% of all the TB patients notified in the region. The DTUs are under the National TB and Leprosy Program (NTLP) and are supported by Doctors with Africa CUAMM.

### Study population

#### Quantitative

Former TB patients diagnosed with drug-susceptible TB (pulmonary bacteriologically confirmed TB (PBC TB), pulmonary clinically diagnosed TB and extra-pulmonary TB) and registered between April 2018 and March 2019 were identified from a review of the unit TB registers. Former TB patients were included if they resided in the study districts, had drug-susceptible TB and provided informed consent. Patients who were diagnosed with rifampicin-resistant and multi-drug resistant TB or had died were excluded from the study. A sample size of 313 participants was calculated using a formula comparing two proportions, assuming a 15-point difference in the treatment success rate for members of the lowest SES quintile from the average rate in Karamoja. This was adjusted by a factor of 1.5 for clustering in DTUs and by 27% for LTFU.

Two line lists for successful and unsuccessful treatment outcomes were generated based on the outcomes assigned to former TB patients using the NTLP guidelines. For this study, successful TB treatment outcomes included cured and treatment completed; unsuccessful outcomes included treatment failed, not evaluated and LTFU. Random computer-generated numbers were used to select 313 participants for the study in a ratio of 1:2 for successful versus unsuccessful TB treatment outcomes. The sample size was distributed across the 10 DTUs proportionally based on the number of patients notified during the study period.

A line list of patients with PBC TB was developed from a review of the laboratory register and this was compared against the unit TB register to identify patients who had not initiated treatment between April 2018 and March 2019 i.e., the pre-treatment LTFU. The registers in Loputuk HC III, Iriiri HC III and Matany Hospital were reviewed. Sputum samples that were referred from other facilities were not included in the analysis.

#### Qualitative

Purposive sampling was used to obtain diversity within each FGD in terms of baseline characteristics and treatment outcomes of former TB patients and with KII in terms of healthcare worker (HCW) cadres. Four FGDs were conducted in two DTUs that experienced high loss to follow-up rates; two in Moroto Regional Referral Hospital and two in Tokora Health Centre IV. To capture health system challenges, 18 KII with HCWs involved in TB diagnosis and treatment were carried out until thematic saturation was reached. These included interviews with community health workers [3], staff at the DTUs [6] and their district health team members [6], regional staff [1] and national supervisors [2].

### Data collection

#### Quantitative

Data were collected on android tablet computers onto which the KoBoCollect app was installed and uploaded onto a corresponding KoBoToolbox electronic database. SES was collected using a questionnaire based on the demographic and health survey wealth index [38]. Wealth was used to measure SES instead of annual income because it captures information on asset ownership which depicts long-term status and can take savings from previous incomes into account. Furthermore, since the majority of Karamoja’s inhabitants are agro-pastoral nomads, income-earning would be subject to seasonal variability. SES data were collected alongside data on socio-demographic characteristics and TB treatment. A separate KoBoToolbox database was developed to collect data on pre-treatment loss to follow-up.

#### Qualitative

Scheduling was done for KIIs at a time and place that was convenient for the participants. FGDs were conducted at the DTU in a secure, relaxed environment and participants recruited from these facilities were given a date on which the discussion would be held. All interviews were recorded and written informed consent was obtained. A local dialect, Ng’akarimojong, was used for the FGDs and English for the KIIs. FGDs were conducted by a social scientist and the KIIs were conducted by a social scientist and a trained field coordinator. The semi-structured interview guide in Additional file 1 was used to steer the discussions during the interviews. The FGD facilitator encouraged the participation of all group members and the expression of divergent experiences and opinions.

### Data analysis

#### Quantitative

Baseline characteristics of study participants were summarised using proportions for categorical variables and median for continuous variables. Principle component analysis was used to analyse data collected on the wealth index including ownership of assets like a motorcycle, computer, television, radio, house, domestic animals, and the type of building materials for the houses they lived in. The wealth index excluded respondents’ occupation and employment status. Wealth quintiles were generated with the upper quintile representing high SES and the lower quintile representing low SES. Treatment outcomes were compared between quintiles using the proportion test. The primary outcome in the study was an unsuccessful treatment outcome and the primary predictor was the SES measured by the wealth index. Participants were also stratified by sociodemographic characteristics and we evaluated their association with TB treatment outcomes using the chi-square test. Variables with a *p*-value less than 0.2 were included in a multivariable analysis using a multiple logistic regression model. Model building was then conducted first, by checking for multicollinearity problems using variance inflation factor (VIF) whereby variables with VIF > 10 were considered to cause multicollinearity. In case of multicollinearity, centring was considered for continuous variables or dropped if categorical and of less biological or statistical significance. Then, a backward elimination process was used to further build the model whereby variables with the biggest *p*-values were dropped one at a time and a likelihood ratio test was used to compare nested models. This process was continued until no further variables could be deleted without a statistically significant loss of fit. Data on pre-treatment loss to follow-up were summarised using proportions. All analyses were done in STATA v14.

Dichotomised TB treatment outcome values were used to generate a concentration curve that showed the cumulative proportion of unsuccessful TB treatment outcomes by the cumulative proportions of individuals in the study population ranked by wealth from poorest to richest. The concentration index was calculated using the formula:

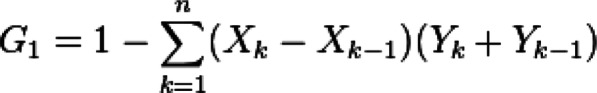



Where G is the coefficient, X is the cumulative proportion of the population variable, Y is the cumulative proportion of the outcome variable and K is the number of individuals. This analysis was done using Microsoft Excel.

Pre-treatment LTFU and their socio-demographic characteristics were summarised using proportions.

#### Qualitative

The FGDs and KIIs were transcribed verbatim. The transcripts from the FGDs were translated to English. The transcripts were analysed using content analysis to identify meaning units that were condensed into codes and categorised into sub-themes and themes. To enhance reliability, the transcripts were analysed separately by two investigators and the themes generated were compared and discussed. Saturation for the KII was analysed by organising the interviews in batches of six and analysing the number of batches needed to generate 80–90% of all codes [43].

#### Data integration

Narrative data integration was done by comparing and contrasting quantitative and qualitative data to evaluate coherence in the study findings [41]. Themes from the qualitative data were compared to corresponding quantitative variables for agreement.

### Ethics

Ethical approval for the study was sought from Mulago Hospital Research and Ethics Committee (MHREC No. 1765) and the Uganda National Council of Science and Technology (HS 2712). Written informed consent was obtained from all participants aged 18 years and above. Parents or legal guardians provided informed consent for minors and assent was obtained from minors aged 8–17 years.

## Results

### Quantitative phase

#### Study participants

**Fig. 1:**
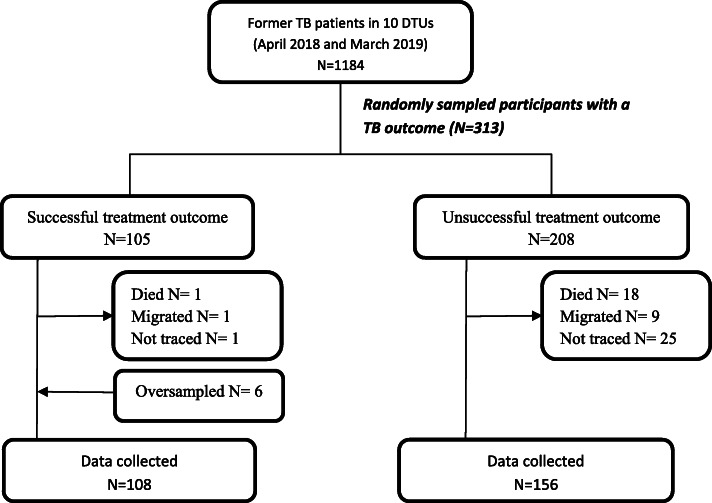
Study participant flow chart

We identified 1184 former TB patients recorded in the unit TB registers and from these, we randomly selected 313 to participate in the study. We oversampled six participants with successful treatment outcomes and requisite notification was given to the ethics committee. Data were collected from 264 (84.3%) participants as shown in Fig. 1.

The sociodemographic characteristics of the participants and their distribution across the wealth index quintiles are presented in Table 1. The study had more males (57.2%) than females (42.8%), and the median age of the participants was 30 years (IQR 13–44). Relative to each variable, the wealthiest quintile had higher proportions of participants who were male (23.2%), aged 25–34 years (32.7%), had three or more meals a day (43.2%) with secondary (81.8%) or tertiary education (100%). They also had the highest proportion of household heads with secondary (70.6%) or tertiary education (100%).

**Table 1:** Distribution of study population sociodemographic characteristics in wealth index quintiles

Variable		Total N	Q1 N (%)	Q2 N (%)	Q3 N (%)	Q4 N (%)	Q5 N (%)
		264	53	53	53	53	52
Sex	Male	151	26 (17.2)	31 (20.5)	27 (17.9)	32 (21.2)	35 (23.2)
	Female	113	27 (23.9)	22 (19.5)	26 (23.0)	21 (18.6)	17 (15.0)
Age	< 15	72	12 (16.7)	27 (37.5)	16 (22.2)	11 (15.3)	6 (8.3)
	15–24	30	9 (30.0)	5 (16.7)	2 (6.7)	9 (30.0)	5 (16.7)
	25–34	55	7 (12.7)	12 (21.8)	11 (20.0)	7 (12.7)	18 (32.7)
	35–44	42	11 (26.2)	3 (7.1)	8 (19.1)	12 (28.6)	8 (19.1)
	45–54	23	3 (13.0)	1 (4.4)	5 (21.7)	9 (39.1)	5 (21.7)
	55–64	18	6 (33.3)	1 (5.6)	4 (22.2)	2 (11.1)	5 (27.8)
	> = 65	24	5 (20.8)	4 (16.7)	7 (29.2)	3 (12.5)	5 (20.8)
HH	Parent	82	16 (19.5)	30 (36.6)	14 (17.1)	13 (15.9)	9 (11.0)
	Spouse	53	15 (28.3)	10 (18.9)	13 (24.5)	8 (15.1)	7 (13.2)
	Respondent	119	22 (18.5)	13 (10.9)	21 (17.7)	32 (26.9)	31 (26.1)
	Other	10	0 (0.0)	0 (0.0)	5 (50.0)	0 (0.0)	5 (50.0)
Respondent’s education level	None	203	42 (20.7)	46 (22.7)	44 (21.7)	43 (21.2)	28 (13.8)
	Primary	46	11 (23.9)	5 (10.9)	9 (19.6)	10 (21.7)	11 (23.9)
	Secondary	11	0 (0.0)	2 (18.2)	0 (0.0)	0 (0.0)	9 (81.8)
	Tertiary	4	0 (0.0)	0 (0.0)	0 (0.0)	0 (0.0)	4 (100)
HH’s education level	No education	209	44 (21.1)	50 (23.9)	43 (20.6)	43 (20.6)	29 (13.9)
	Primary	33	8 (24.2)	2 (6.1)	9 (27.3)	8 (24.2)	6 (18.2)
	Secondary	17	1 (5.9)	1 (5.9)	1 (5.9)	2 (11.8)	12 (70.6)
	Tertiary	5	0 (0.0)	0 (0.0)	0 (0.0)	0 (0.0)	5 (100)
Respondent’s occupation	None	73	13 (17.8)	26 (35.6)	21 (28.8)	8 (11.0)	5 (6.9)
	Subsistence farmer	146	32 (21.9)	22 (15.1)	27 (18.5)	35 (24.0)	30 (20.6)
	Formal	7	0 (0.0)	0 (0.0)	0 (0.0)	0 (0.0)	7 (100)
	Informal	19	1 (5.3)	2 (10.5)	4 (21.1)	4 (21.1)	8 (42.1)
	Student	19	7 (36.8)	3 (15.8)	1 (5.3)	6 (31.6)	2 (10.5)
Employed	Yes	50	1 (2.0)	4 (8.0)	10 (20.0)	15 (30.0)	20 (40.0)
	No	158	40 (25.3)	25 (15.8)	33 (20.9)	31 (19.6)	29 (18.4)
	Not applicable^ɣ^	56	12 (21.4)	24 (42.9)	10 (17.9)	7 (12.5)	3 (5.4)
HH’s occupation	None	20	7 (35.0)	2 (10.0)	5 (25.0)	5 (25.0)	1 (5.0)
	Subsistence farmer	206	46 (22.3)	47 (22.8)	40 (19.4)	42 (20.4)	31 (15.1)
	Formal	13	0 (0.0)	1 (7.7)	0 (0.0)	2 (15.4)	10 (76.9)
	Informal	25	0 (0.0)	3 (12.0)	8 (32.0)	4 (16.0)	10 (40.0)
HH employed	Yes	67	2 (3.0)	7 (10.5)	13 (19.4)	21 (31.3)	24 (35.8)
	No	195	51 (26.2)	46 (23.6)	39 (20.0)	31 (15.9)	28 (14.4)
	Not applicable^ɣ^	2	0 (0.0)	0 (0.0)	1 (50.0)	1 (50.0)	0 (0.0)
Marital status^ɸ^	Single	33	5 (15.2)	8 (24.2)	5 (15.2)	5 (15.2)	10 (30.3)
	Married	131	30 (22.9)	19 (14.5)	26 (19.9)	30 (22.9)	26 (19.9)
	Divorced/separated	15	4 (26.7)	1 (6.7)	1 (6.7)	4 (26.7	5 (33.3)
	Widowed	21	4 (26.7)	2 (9.5)	6 (28.6)	4 (19.1)	5 (33.3)
Meals per day	One	90	20 (22.2)	21 (23.3)	18 (20.0)	19 (21.1)	12 (13.3)
	Two	137	30 (21.9)	30 (21.9)	32 (23.4)	21 (15.3)	24 (17.5)
	Three or more	37	3 (8.1)	2 (5.4)	3 (8.1)	13 (35.1)	16 (43.2)
Meal satisfaction	Yes	152	12 (7.9)	31 (20.4)	32 (21.2)	39 (25.7)	38 (25.0)
	No	112	41 (36.6)	22 (19.6)	21 (18.8)	14 (12.5)	14 (12.5)
Alcohol consumption	Yes	130	26 (20.0)	22 (16.9)	27 (20.8)	27 (20.8)	28 (21.5)
	No	134	27 (20.2)	31 (23.1)	26 (19.4)	26 (19.4)	24 (17.9)

ɣ Not applicable was defined as participants under the age of 15 who were not in school or employed

ɸ Marital status was not evaluated for participants under 15 who were not married, divorced/separated or widowed

HH Household head

### TB treatment outcomes by wealth

The treatment outcomes of participants by wealth are presented in Table 2. A total of 156 participants (59.1%) had unsuccessful treatment outcomes. The wealthiest quintile had significantly lower odds of having an unsuccessful treatment outcome (OR = 0.41, 95% CI: 0.19–0.90, *p* = 0.026).

**Table 2:** Impact of wealth index quintiles on TB treatment outcomes

Wealth index quintiles	Unsuccessful treatment N (%)	Successful treatment N (%)	Unadjusted O. R (95% CI)	*p*-value	Adjusted OR (95% CI)	*p*-value
Q1 (Poorest reference)	33 (21.2)	20 (18.5)	1		1	
Q2	34 (21.8)	19 (17.6)	1.08 (0.49–2.39)	0.840	0.96 (0.42–2.19)	0.920
Q3	34 (21.8)	19 (17.6)	1.08 (0.49–2.39)	0.840	1.12 (0.49–2.54)	0.794
Q4	34 (21.8)	19 (17.6)	1.08 (0.49–2.39)	0.840	1.06 (0.46–2.41)	0.894
Q5 (richest)	21 (13.5)	31 (28.7)	0.41 (0.19–0.90)	**0.026**	0.42 (0.18–0.99	**0.047**

**Fig. 2:**
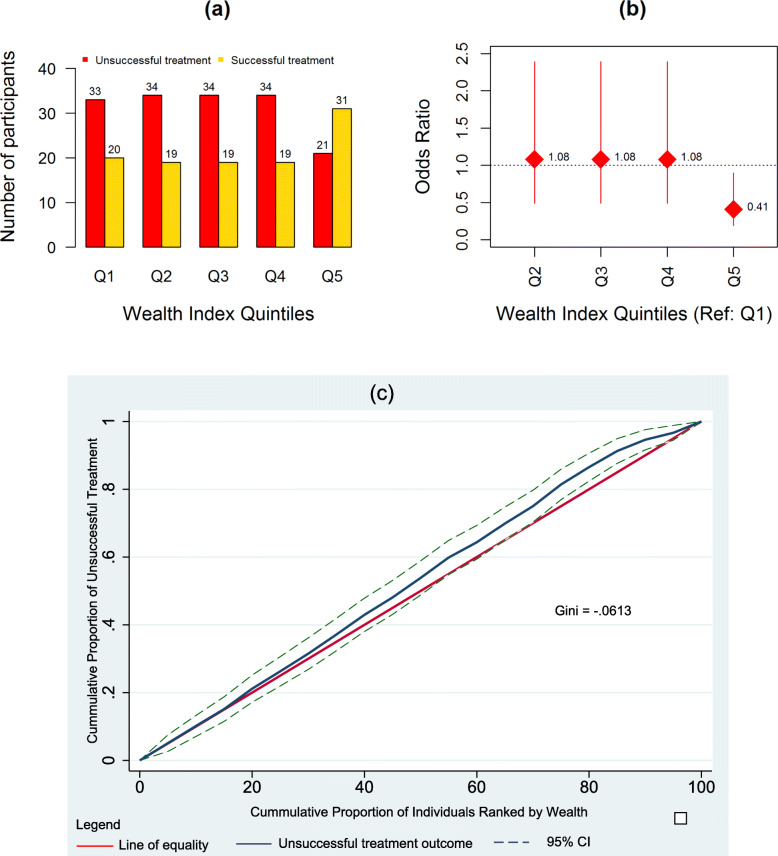
Treatment outcomes by wealth. **a** Treatment outcomes by wealth index quintiles. **b** Odds ratios by wealth index quintiles. **c** Concentration curve for unsuccessful treatment outcomes

Figure 2 shows that 40.4% (21/52) of the participants in the highest wealth quintile had unsuccessful treatment outcomes with higher proportions of unsuccessful treatment outcomes observed in the lower four wealth quintiles: 62.3% (33/53) in Q1 and 64.2% (34/53) in Q2-Q4. The concentration curve shows that the distribution of unsuccessful treatment outcomes in individuals ranked by wealth from poorest to richest was pro-poor. A negative concentration index of −0.061 was computed.

Due to homogeneity in the lower four quintiles, a secondary analysis was performed on the distribution of participants by sociodemographic characteristics and SES with the lower four wealth quintiles (poor) compared to the highest quintile (rich). The odds of being in the highest wealth quintile were higher for participants aged 25–34 years (OR = 5.35, 95% CI 1.95–14.66, *p* = 0.001) and 55–64 years (OR = 4.23, 95% CI 1.12–15.96, *p* = 0.033). Secondary education of the respondent and household head was also strongly associated with being in the wealthiest quintile with odds ratios of 28.12 (95% CI 5.77–136.99, *p* < 0.001) and 14.9 (95% CI 4.88–45.41, p < 0.001) respectively. All participants who had tertiary education belonged to the highest wealth quintile. Participants who had three meals or more or reported satisfaction after a meal had higher odds of being in the highest quintile at 4.95 (95% CI 2.03–12.06, *p* < 0.001) and 2.33 (95%CI 1.19–4.56, *p* = 0.013). Marital status and alcohol consumption was not significantly associated with belonging to the highest wealth quintile while mixed results were obtained on the nature of employment. Full results are in Additional file 2.

### TB treatment outcomes by socio-demographic characteristics

The impact of socio-demographic characteristics on TB treatment outcomes is presented in Table 3. The odds of having an unsuccessful treatment outcome were significantly lower for formally employed household heads (OR = 0.19, 95% CI: 0.04–0.87, *p* = 0.032). The participants’ treatment outcome was not significantly associated with gender, age, nature of household head, respondent’s occupation, respondent’s or household head’s level of education, marital status, meal frequency, meal satisfaction and alcohol consumption. Regarding HIV, 4.5% of the TB patients were co-infected with HIV. A positive HIV status was not significantly associated with unsuccessful treatment outcomes (OR = 0.97, 95% CI 0.30–3.13, *p* = 0.956).

**Table 3:** Distribution by sociodemographic characteristics and TB treatment outcomes

Variable		Unsuccessful treatment N (%)	Successful treatment N (%)	O.R (95% CI)	p-value
Sex	Male	86 (55.1)	65 (60.2)	1	
	Female	70 (44.9)	43 (39.8)	1.23 (0.75–2.02)	0.414
Age	< 15	46 (29.5)	26 (24.1)	1	
	15–24	21 (13.5)	9 (8.3)	1.32 (0.53–3.30)	0.554
	25–34	27 (17.3)	28 (25.9)	0.55 (0.27–1.11)	0.096
	35–44	24 (15.4)	18 (16.7)	0.75 (0.35–1.64)	0.476
	45–54	13 (8.3)	10 (9.3)	0.73 (0.28–1.91)	0.527
	55–64	7 (4.5)	11 (10.2)	0.36 (0.12–1.04)	0.059
	> = 65	18 (11.5)	6 (5.6)	1.70 (0.60–4.81)	0.320
HH	Parent	51 (32.7)	31 (28.7)	1	
	Spouse	32 (20.5)	21 (19.4)	0.93 (0.46–1.88)	0.832
	Respondent	67 (43.0)	52 (48.2)	0.78 (0.44–1.39)	0.405
	Other	6 (3.9)	4 (3.7)	0.91 (0.24–3.49)	0.893
Respondent’s education level	No education	122 (78.2)	81 (75.0)	1	
	Primary	28 (18.0)	18 (16.8)	1.03 (0.53–1.99)	0.923
	Secondary	4 (2.6)	7 (6.5)	0.38 (0.11–1.34)	0.132
	Tertiary	2 (1.3)	2 (1.9)	0.66 (0.09–4.81)	0.685
HH’s education level	No education	129 (82.7)	80 (74.1)	1	
	Primary	18 (11.5)	15 (14.0)	0.74 (0.36–1.56)	0.434
	Secondary	7 (4.5)	10 (9.3)	0.43 (0.16–1.19)	0.104
	Tertiary	2 (1.3)	3 (2.8)	0.41 (0.07–2.53)	0.339
Respondent’s occupation	None	43 (27.6)	30 (27.8)	1	
	Subsistence farmer	86 (55.1)	60 (55.6)	1.00 (0.56–1.77)	> 9.999
	Formal	1 (0.6)	6 (5.6)	0.12 (0.01–1.02)	0.052
	Informal	14 (9.0)	5 (4.6)	1.95 (0.64–6.00)	0.242
	Student	12 (7.7)	7 (6.5)	1.20 (0.42–3.39)	0.736
Respondent’s employment	No	89 (57)	69 (63.9)	1	
	Yes	28 (18.0)	22 (20.4)	0.99 (0.52–1.97)	0.967
	Not applicable^ɣ^	39 (25.0)	17 (15.7)	1.78 (0.93–3.41)	0.083
HH’s occupation	None	14 (9.0)	6 (5.6)	1	
	Subsistence farmer	122 (78.2)	84 (77.8)	0.62 (0.23–1.69)	0.351
	Formal	4 (2.6)	9 (8.3)	0.19 (0.04–0.87)	**0.032**
	Informal	16 (10.3)	9 (8.3)	0.76 (0.22–2.68)	0.672
HH employment	No	119 (76.3)	76 (70.4)	1	
	Yes	36 (23.1)	31 (28.7)	0.74 (0.42–1.30)	0.295
	Not applicable^ɣ^	1 (0.6)	1 (0.9)	0.64 (0.04–10.36)	0.752
Marital status	Single	20 (12.8)	13 (12.0)	1	
	Married	72 (46.2)	59 (54.6)	0.79 (0.36–1.73)	0.560
	Divorced/ separated	11 (7.1)	4 (3.7)	1.79 (0.47–6.83)	0.396
	Widowed	13 (8.3)	8 (7.4)	1.06 (0.34–3.25)	0.924
Meals per day	One	51 (32.7)	39 (36.1)	1	
	Two	88 (56.4)	49 (45.4)	1.37 (0.80–2.37)	0.253
	Three	17 (10.9)	20 (18.5)	0.65 (0.30–1.40)	0.272
Meal satisfaction	No	71 (45.5)	41 (38.0)	1	
	Yes	85 (54.5)	67 (62.0)	0.73 (0.44–1.21)	0.223
Alcohol consumption	No	84 (53.9)	50 (46.3)	1	
	Yes	72 (46.2)	58 (53.7)	0.74 (0.45–1.21)	0.228
HIV status	Negative	149 (95.5)	103 (95.4)	1	
	Positive	7 (4.5)	5 (4.6)	0.97 (0.30–3.13)	0.956

ɣ Not applicable was defined as participants under the age of 15 who were not in school or employed

ɸ Marital status was not evaluated for participants under 15 who were not married, divorced/separated or widowed

HH Household head

For the multivariable analysis, the final statistical model included the following variables: wealth index quintiles, occupation and employment status. Results from the final model are presented as adjusted estimates and the goodness of fit test showed borderline fit (*p* = 0.063). The final fit was free from multicollinearity with mean VIF values of 1.7 for wealth index and occupation, and 1.9 for employment. Table 4 shows the results from the better fit. The adjusted odds of unsuccessful TB treatment outcome in the highest wealth quintile were significantly lower compared to the poorest quintile (OR = 0.42, 95% CI 0.18–0.9, p = 0.047). Odds of unsuccessful treatment outcome were higher among participants employed in the informal sector (OR = 4.71, 95% CI 1.18–18.89, p = 0.029) and children under the age of 15 years who were not in school or employed (OR = 2.71, 95% CI 1.11–6.62, p = 0.029).

**Table 4:** Unadjusted and adjusted odds ratios for factors associated with unsuccessful treatment outcomes

Variable	Unadjusted OR (95% CI)	*p*-value	Adjusted OR (95% CI)	*p*-value
**Wealth index quintiles**				
Q1 (Poorest reference)	1		1	
Q2	1.08 (0.49–2.39)	0.840	0.96 (0.42–2.19)	0.920
Q3	1.08 (0.49–2.39)	0.840	1.12 (0.49–2.54)	0.794
Q4	1.08 (0.49–2.39)	0.840	1.06 (0.46–2.41)	0.894
Q5 (richest)	0.41 (0.19–0.90)	**0.026**	0.42 (0.18–0.99	**0.047**
**Respondent’s occupation**				
None	1		1	
Subsistence farmer	1.00 (0.56–1.77)	> 9.999	1.92 (0.90–4.10)	0.091
Formal	0.12 (0.01–1.02)	0.052	0.45 (0.04–5.11)	0.523
Informal	1.95 (0.64–6.00)	0.242	4.71 (1.18–18.89)	**0.029**
Student	1.20 (0.42–3.39)	0.736	1.05 (0.34–3.22)	0.936
**Respondent’s employment**				
No	1		1	
Yes	0.99 (0.52–1.97)	0.967	1.01 (0.45–2.28)	0.986
Not applicable^ɣ^	1.78 (0.93–3.41)	0.083	2.71 (1.11–6.62)	**0.029**

ɣ Not applicable was defined as participants under the age of 15 who were not in school or employed

### Healthcare system factors and TB treatment outcomes

Additional file 3 shows the analysis of healthcare system factors by TB treatment outcomes. Under half (48.7%) of all patients reported that they lived within 5kms from the healthcare unit, an almost equal proportion (51.3%) rated the distance to the health facility as long. Generally, the participants were satisfied with healthcare services received at 96.8% for participants with unsuccessful treatment and 96.3% for participants with successful treatment. These factors were not significantly associated with TB treatment outcomes.

### Pre-treatment LTFU

A line list of 256 patients with PBC TB was developed from the laboratory registers and this was compared against the unit TB registers. By sex, there were more male PBC TB patients (141/256, 55.1%) than females. The proportion of pre-treatment LTFU was 17.2% and this proportion was higher in females than males at 21.7% (25/115) versus 13.5% (19/141). By facility, Matany Hospital had the highest pre-treatment LTFU rate at 22.8% followed by Iriiri HC III at 9.1%. No pre-treatment LTFUs were identified at Loputuk HC III.

### Qualitative phase

#### Treatment barriers and motivators

Analysis of the KIIs generated 46 codes and 89.1% (41/46) of the codes were generated in the first batch of six interviews. Saturation was reached in the second batch of interviews. Two major thematic areas of barriers and motivators experienced during TB treatment were developed from the analysis of the FGDs and KIIs with the results summarised in Table 5 below.

**Table 5:** Barriers and motivators during TB treatment

Dimension	Barrier	Motivator
**Intrapersonal**	Shortage of money	Perceived benefits of taking medication
	Lack of food to eat before taking medication	
**Interpersonal**	Stigma and the fear of being stigmatized	Social support from family and friends such as food, money, bedside care
		Treatment support from HCWs like counselling, follow-up calls
**Environmental**	Migration in search of food, pasture and water for animals, mining and porous borders	Health system and community support by NGOs
	Difficulties in transportation due to limited modes of transport, long travel distances and weather	Adapting service delivery mechanisms to meet needs through teamwork and internal reorganization
	Drug and equipment stock-outs	Strengthening lower-level facilities through support supervision, mentorship, capacity building and training
	Lack of access to financial support while sick to meet basic needs	Community engagement through community sensitization, radio talk shows and directly at the health facilities

#### TB treatment barriers

TB patients reported that they did not have adequate finances to meet their basic needs including food and transport during TB treatment. Participants reported that they looked for menial jobs, sold assets such as farm animals or relied on family and friends for financial support during treatment.

*There is no way you can get money to board a motorcycle, so you come on foot to get your drugs and when you return, you go and look for a job to get something to cook.* [Female FGD participant]

As a result of financial constraints, participants lacked adequate food to meet their nutritional needs. Both former TB patients and HCWs used different expressions to demonstrate the perception that adequate nutrition is needed during TB treatment. Participants reported that they preferred to take their medications after a meal, usually milk or porridge, with some reporting that they did not take their medication if they failed to get food. Notably, patients reported that they had no sources of financial aid while sick and appealed for support, particularly from the government in terms of food

*What we have to say is that you as government please rescue us in terms of some food because every day what we eat is the tree leaf that is why this disease has refused to go because of hunger.* [Male FGD participant]

The demand to make a living while on treatment contributed to poor treatment outcomes. TB patients in the region were noted to be highly mobile and nomadic, often migrating from their usual place of residence. This was attributed to economic activities like agriculture and mining. HCWs acknowledged that LTFU is a major problem in the Karamoja region due to the mobile communities that made follow-up tedious. The porous borders, particularly with Kenya, made it easy for patients to get LTFU during treatment. Some patients also moved deliberately ‘*as a way of dodging drugs.’* Only one formally employed participant reported sick leave for the duration of TB treatment.

*We have also found that there is a lot of mobility among the patients ( … ) the moment you are to continue on treatment, they have disappeared. This is a nomadic area; they will have disappeared to another place and finding them may be difficult.* [Male HCW]

Limited modes of transport within the region and long distances were highlighted as challenges for both the HCWs and TB patients in delivering and accessing healthcare services respectively. HCWs reported that difficulties in accessing transport was not only detrimental to service delivery but also demotivated them. Transport difficulties affected services such as providing support supervision, updating registers, fieldwork activities, transporting TB samples to designated units and transporting TB results.

*( … ) it a very long distance even if you went in a car you will reach late. When I came back here, it was a Sunday and there were no nurses so I just relaxed until the TB came back ( … )* [Female FGD participant]

*( … ) they need to offer transport for us. We are ready to work at any time, whenever we are called to do any work.* [Female HCW]

TB patients reported experiencing stigma and abandonment from family members. Stigma and the fear of being stigmatised influenced patient’s behaviour before, during and after the TB treatment. One participant decided to relocate during treatment because of stigma while another was driven to seek treatment far away from her home as quoted below:

*All the people who were mine feared me; even my own mother feared me, all the family members fear me so when I saw that everyone was fearing me, I went up to Tokora on foot carrying my baby on the back.* [Female FGD participant]

Former TB patients described experiences in which they missed drugs because they were not available from the facility. HCWs also noted that although there has been an improvement in the supply of laboratory equipment and drugs used in managing TB, occasionally they experienced stock-outs which resulted in LTFU particularly among paediatric patients. Equipment challenges included GeneXpert cartridges stock-out, breakdown of microscopes and X-ray machines and power shortages despite local solar power systems leading to difficulties in correctly diagnosing and following up TB patients.

*Then the other challenging part is the stock-out of drugs. ( … ) Last December we had medicines for adults, but the problem, we did not have paediatric formulations for TB treatment: the whole December, we did not have anything.* [Male HCW]

#### TB treatment motivators

Social and economic support was mentioned as a motivator for treatment adherence. Patients noted that they received both tangible and non-tangible support such as money, food and bedside care. Female relatives such as wives and grandmothers often offered care and support to TB patients. HCWs also provided treatment support for patients like offering counselling and reminder phone calls about upcoming refill appointments, which were beneficial for treatment adherence and completion.

*I did not have money. If my wife went to sell charcoal, she would bring for me. When she hasn’t gone then I also don’t have.* [Male FGD participant]

*Yeah, for me the other factor that made it possible to come for my appointments was reminders, at least I had reminders most of the times.* [Male FGD participant]

Former TB patients noted that after initiating treatment, their symptoms started to subside and for some, this motivated them to continue treatment. On the other hand, the improvement experienced while on treatment made some patients stop taking treatment as they believed they were cured.

*The good thing about that drug is that when you take it you stop coughing.* [Male FGD participant]

The HCWs noted that both the government and non-governmental organisations (NGOs) are working in the region to support TB programs. This collaboration has been credited for improving the quality, efficiency and reach of TB services particularly through community dialogues. Community engagement through which health education is delivered either through community sensitization meetings, radio talk shows or at health facility entry points is credited for improvements in TB treatment. These efforts were intensified after identifying Karamoja as a TB ‘hot spot’ and realizing that some TB patients often fail to complete. HCWs also credited adaptions to services delivery at the health facility to improvements in TB care. One noted that the number of LTFU cases was higher in 2018 than in 2019 and credited this drop to teamwork and adopting a task-shifting approach in which TB drug refills were carried out by any staff member on the TB unit.

*One of it is high collaboration with partners. Partners have been helping us too much ( … ) we have always moved with them to mentor our staff, we also do contact tracing with them, we have also distributed food that was given by OPM [0ffice of the Prime Minister] government to our district to make sure that our clients, TB AND HIV clients, benefit from it.* [Male HCW]

Support supervision, mentorship, capacity building and training were credited for strengthening lower-level facilities and a reduction in treatment default rate. Support supervision through a top-down demand for accountability was seen as a beneficial process by both the supervisors and the HCWs at the facilities. It also served as a tool to address challenges in motivation and attitudes to providing TB care.

#### Data integration

Quantitative and qualitative data were compared and converged. The quantitative data showed that poor quintiles had higher proportions of unsuccessful treatment outcomes and this corroborated interview findings in which patients highlighted the difficulty in getting food and transport brought on by financial hardship during TB treatment. Although the number of meals per day and meal satisfaction was not independently associated with treatment outcomes, HCWs and patients’ perceptions were that food was important during TB treatment, affecting treatment adherence. Despite the lack of a direct association, quantitative data showed that high meal frequency and meal satisfaction was associated with the wealthiest quintile. Formal employment of the household head was protective for unsuccessful treatment outcomes (univariate analysis) and informal employment was positively associated with unsuccessful treatment outcomes (multivariable analysis). Participants reported that they continued to work during treatment and the only participant that got sick leave was formally employed.

Both quantitative and qualitative data showed high levels of satisfaction with the healthcare services with treatment support such as counselling and follow-up calls identified as beneficial during treatment. However, health services delivery was affected by drug and equipment stock-outs, long travel distances and few modes of transport.

## Discussion

Regarding TB, Karamoja is a high burden region in a high burden country. While efforts have been made by the health system to detect all TB cases in the region, there is still a very low proportion of patients with successful treatment outcomes in TB care. This study, done in this poor rural region in Uganda [37, 38], found a significant association between SES and TB treatment outcomes. Having a high wealth quintile, in our experience, reduces the risk of an unsuccessful treatment outcome by 58%. A vast majority of the modifiable risk factors for TB are linked to poverty and low SES. Poor TB outcomes in this region were attributed in part to the poor socio-economic status, rampant poverty and high levels of illiteracy.

The wealthiest quintile had significantly lower odds of having an unsuccessful treatment outcome and these findings are in line with previous studies [15]. It is clear that the effect of wealth on TB treatment outcomes significantly manifests only in the wealthiest quintile. Arguably, the majority of Karamoja’s inhabitants are homogenously poor [37, 38], and this may explain the similar TB treatment outcomes observed in the lower quintiles, initially paralleling the line of equality.

Socio-demographic characteristics related to poverty have their independent effects on treatment outcomes such as the type of employment. The odds of having poor treatment outcomes were higher for individuals employed in the informal sector; conversely, formal employment of household heads independently reduced the odds of having poor treatment outcomes. Being a child below 15 and not attending school was similarly detrimental to successful treatment for TB. Our results align with findings reported in other settings. For instance, a South African study evaluating TB mortality found that it varied across occupation groups with the highest rates observed among people in an elementary occupation such as agricultural workers, cleaners and refuse workers [44]. Although other sociodemographic factors were not found to be associated with treatment outcome in this study, other studies have found the level of education to be significantly associated with it [17]. The wealth quintile effect on treatment outcomes may indirectly work through the level of education as individuals in this study belonging to the highest wealth quintile were more likely to have had secondary or tertiary education.

Study participants had a preference for or believed that TB drugs should be taken after meals and subsequently, many reported not taking them if they failed to get food. In a place like Karamoja that often experiences food scarcity, one can understand why many patients are discontinuing treatments. This phenomenon is not something new as previous studies already showed lack of food, stigma, lack of social and economic support during treatment and long distances to health facilities affected patients’ treatment adherence [45–47]. Financial challenges during TB treatment do impact patients’ ability to cater for food and transport costs during treatment. These challenges are exacerbated by the high mobility of the population, especially across the Uganda-Kenya border for economic reasons, which makes treatment follow-up difficult.

Patients in this study relied on family or friends as a social support system to meet their financial expenditures and loss of income and did not report access to any organised social protection schemes. Amid low levels of social protection and limited savings, the poorest patients are more likely to resort to coping mechanisms such as the sale of assets or borrowing to defray costs plunging them further into poverty [48]. Poverty and TB are intrinsically intertwined as poverty fosters TB and, in turn, TB can lead to loss of income and the costs of managing TB can become catastrophic [49–54]. A survey done in Uganda showed that over half of TB patients experienced catastrophic costs [48] and these have been linked to poor outcomes [55].

Globally, we are not on track to meet the milestones of the End TB strategy for 2025. The current TB case fatality ratio of 14% should fall to about 7% and the annual 1.7% reduction in TB incidence rate is only a factor of the requisite 10% drop rate [1]. Integrated, patient-centered care and prevention are crucial to ending TB as a public health problem and models have shown that interventions to end extreme poverty and expand social protection coverage alone can result in a significant decline in TB incidence and protect people with TB from untoward financial hardships including catastrophic costs [30]. People living in socio-economically deprived areas face higher TB mortality [56, 57] and population-level interventions like increased government spending on social protection initiatives have been shown to reduce TB incidence and mortality [58–60]. Social protection programmes such as conditional cash transfer payments linked to TB treatment targets or microfinance schemes have been shown to reduce vulnerability, alleviate poverty, and improve food security and treatment outcomes [14, 49, 61–63]. Social protection schemes that address the broader determinants of health and protect against tuberculosis-associated financial consequences are needed to improve the treatment outcomes in the Karamoja region.

In Karamoja, HIV prevalence is lower than the national average [64] and as such, HIV is not a major determinant of TB and outcomes: only 4.5% of the study participants were HIV positive. In contrast to other studies [17, 20] that had a higher HIV co-infection rate, HIV did not increase the risk of having an unsuccessful TB treatment outcome in this study.

This study has some limitations. First of all, there is a selection bias due to the 1:2 randomization design between successful and unsuccessful outcomes. If poor TB treatment outcomes are generally linked to low wealth quintiles, there may have been an increased chance of selection of those belonging to the real-life poorer wealth quintiles. As a result, a high proportion of the sample especially of people in Q1–4 may have clustered in the lower ‘real-life’ wealth quintiles. This might explain why significantly lower odds of having an unsuccessful treatment outcome were got in only the wealthiest quintile. Secondly, being a retrospective study on patients who had completed treatment, the findings may be subject to recall bias. However, the use of a wealth index to measure SES as opposed to income was of benefit to the study. In contrast to an individual’s income that is limited to a narrow time frame and subject to seasonal variability particularly in the informal sector, wealth is a long-term indicator of SES as it depicts assets accumulated over time, incorporating the impacts of education, employment, income and savings [65, 66]. Another limitation is that patients who died during TB treatment were not included in the study, an additional 19 participants died before the inception of this study. TB mortality is a pertinent indicator in most TB programs and one of the most researched treatment outcomes but we were not able to analyse it in this study. We postulate that patients who died may have had lower SES [57, 67] and this would have increased the significance of our findings. Lastly, patients experiences and perspectives on TB treatment barriers and motivators were explored using FGDs only. Whereas lived experiences are better explored with one-on-one interviews, FGDs were chosen to identify cross-cutting themes and general problems and this may have led to a loss of information on unique, personal experiences.

## Conclusion

This study shows that higher socio-economic status, measured by wealth and related attributes of formal employment, is associated with better TB treatment outcomes. High losses to follow-up before treatment initiation and during the continuation phase are likely affected by lack of food, stigma, belonging to nomadic communities, and lack of transport and finances to meet basic needs. Initiatives that target poverty and other social determinants of health have the potential to accelerate progress to the End TB targets firstly by protecting against TB-associated catastrophic costs and in the long run, reducing TB incidence and mortality. Improving treatment outcomes in Karamoja will require addressing these socio-economic determinants alongside improvements in general programmatic performance. Institution of treatment enablers for all TB patients including provision of food, transport and innovative micro-financing mechanisms is necessary for a poverty-stricken region like Karamoja and should be a priority for health authorities.

## Supplementary Information

**Additional file 1.** Interview Guides. Interview guides used for key informant interviews of health care workers and focus group discussions among former TB patients.

**Additional file 2.** Distribution by sociodemographic characteristics and wealth. Table of results.

**Additional file 3.** Healthcare system factors and TB treatment outcomes. Table of results.

## Data Availability

The datasets used and/or analysed during the current study are available from the corresponding author on reasonable request.

## Abbreviations

DTUs: TB Diagnostic &Treatment Units
FGDs: Focus group discussions
HCW: Healthcare worker
KII: Key informant interviews
LTFU: Loss to follow-up
NGOs: Non-governmental organisations
NTLP: National Tuberculosis and Leprosy Control Program
PBC TB: Pulmonary bacteriologically confirmed TB
SDGs: United Nations Sustainable Development Goals
SES: Socio-economic status
TB: Tuberculosis
