# Supplementary material for: Impact of socio-economic factors on Tuberculosis treatment outcomes in north-eastern Uganda: a mixed methods study
Source: BMC Public Health. 2021 Nov 26;21:2167. doi: 10.1186/s12889-021-12056-1 (PMC8620143; doi:10.1186/s12889-021-12056-1)
Supplement: Supplementary file 1 — Additional file 1. Interview Guides. Interview guides used for key informant interviews of health care workers and focus group discussions among former TB patients. [file 12889_2021_12056_MOESM1_ESM.docx]

**Additional file 1:** **Interview Guides**

1. **Healthcare worker interview guide**

**Introduction:**

Dear participants. I am ……………………... (facilitator introduces him/herself by name) with my colleague …………………… (introduces the note taker/recorder). Makerere Lung Institute (MLI) in collaboration with National Tuberculosis and Leprosy Program (NTLP) at the Ministry of Health and Doctors with Africa CUAMM are carrying out a study to assess the impact of socio-economic factors on the TB outcomes among TB patients in Karamoja, Uganda. The purpose of this discussion is to get a better understanding of the health system factors that facilitated or hindered your work in delivering care to TB patients. With your consent, we will record this discussion on a tape recorder, and we will take notes of whatever is being said. Unless you have any questions, I will start the discussion.

**Health system factors that were beneficial in delivering TB care and treatment:**

1. Please briefly describe your role in the management of TB
2. Please let us know what factors made it possible for you to be able to deliver TB care to patients?
3. Please describe any changes between April 2018- March 2019 that have improved the way you deliver TB care
4. Please describe any factors that motivate you in your work?

**Health system factors that have hindered delivery TB care and treatment:**

1. Please let us know what factors made it challenging for you to be able to deliver TB care to patients?
2. Please describe any changes between April 2018- March 2019 that have made it challenging for you deliver TB care
3. Have you experienced any stock outs or equipment break down between April 2018- March 2019? Please describe how this affected your at work

**General health system factors**

1. Kindly describe how your facility is supervised/ how you supervise health facilities in your district and how this impacts the quality of TB care.
2. Kindly describe how your facility/ district maintains TB records and how this data is useful to your facility/district. Do you analyze and discuss your data?
3. Is there anything else you would like to share with us?

Thank you for your time.

1. **FGD topic guide**

**Introduction:**

Dear participants. I am ……………………... (facilitator introduces him/herself by name) with my colleague …………………… (introduces the note taker/recorder). Makerere Lung Institute (MLI) in collaboration with National Tuberculosis and Leprosy Program (NTLP) at the Ministry of Health and Doctors with Africa CUAMM are carrying out a study to assess the impact of socio-economic factors on the TB outcomes among TB patients in Karamoja, Uganda. The purpose of this discussion is to get a better understanding of the factors that enabled you to adhere to your treatment and/or the social challenges you faced during your TB treatment. Each of you will be given an opportunity to share their opinion by responding to the topics or question that will be raised for discussion. With your consent, we will record this discussion on a tape recorder, and we will take notes of whatever is being said.

Unless someone has any questions, I will start the discussion.

**Social economic factors that were beneficial during TB treatment:**

1. Please briefly describe your TB treatment experience
2. How did your family members, workmates or bosses, friends relate/interact with you as a TB patient that was beneficial to your treatment?
3. How did healthcare workers relate/interact with you as a TB patient that was beneficial to your treatment?
4. Describe the factors at your home and in your community that made it possible for you to take your treatment every day? Tell us about the things that you did before taking your medication?
5. How was your current financial situation as a TB patient beneficial to your treatment?
6. Please let us know what factors made it possible for you to be able to go to the hospital for your appointments?
7. What motivated you to take your treatment?

**Social economic factors that posed challenges during TB treatment:**

1. How did your family members, workmates or bosses, friends relate/interact with you as a TB patient that made your TB treatment difficult?
2. How did healthcare workers relate/interact with you as a TB patient that that made your TB treatment difficult?
3. Describe the factors at your home and in your community that made it difficult for you to take your treatment every day?
4. How was your current financial situation as a TB patient made your treatment difficult?
5. Please let us know what factors made it difficult for you to go to the hospital for all your appointments?
6. What discouraged you from taking your treatment?
7. Is there anything else you would like to share with us?

Thank you for your time.
