# Supplementary material for: Impact of socio-economic factors on Tuberculosis treatment outcomes in north-eastern Uganda: a mixed methods study
Source: BMC Public Health. 2021 Nov 26;21:2167. doi: 10.1186/s12889-021-12056-1 (PMC8620143; doi:10.1186/s12889-021-12056-1)
Supplement: Supplementary file 2 — Additional file 2. Distribution by sociodemographic characteristics and wealth. Table of results. [file 12889_2021_12056_MOESM2_ESM.docx]

**Additional file 2: Distribution by sociodemographic characteristics and wealth**

| Variable | | Poor N (%) | Rich N (%) | O.R (95% CI) | p-value |
| --- | --- | --- | --- | --- | --- |
| Sex | Male | 116 (54.7) | 35 (67.3) | 1 |  |
|  | Female | 96 (45.3) | 17 (32.7) | 0.59 (0.31 - 1.11) | 0.102 |
| Age | <15 | 66 (31.1) | 6 (11.5) | 1 |  |
|  | 15-24 | 25 (11.8) | 5 (9.6) | 2.2 |  |
|  | 25-34 | 37 (17.5) | 18 (34.6) | 5.35 (1.95 – 14.66) | **0.001** |
|  | 35-44 | 34 (16.0) | 8 (15.4) | 2.59 (0.83 – 8.06) | 0.101 |
|  | 45-54 | 18 (8.5) | 5(9.6) | 3.06 (0.84 -11.12) | 0.091 |
|  | 55-64 | 13 (6.1) | 5 (9.6) | 4.23 (1.12 – 15.96) | **0.033** |
|  | >=65 | 19 (8.7) | 5 (9.6) | 2.89 (0.80 -10.54) | 0.107 |
| HH | Parent | 73 (34.4) | 9 (17.3) | 1 |  |
|  | Spouse | 46 (21.7) | 7 (13.5) | 1.23 (0.43 – 3.54) | 0.696 |
|  | Respondent | 88 (41.5) | 31 (59.6) | 2.86 (1.28 – 6.39) | **0.011** |
|  | Other | 5 (2.4) | 5 (9.6) | 8.11 (1.96 – 33.55) | **0.004** |
| Respondent’s education level | No education | 175 (82.6) | 28 (53.9) | 1 |  |
|  | Primary | 35 (16.5) | 11 (21.2) | 1.96 (0.89 – 4.31) | 0.092 |
|  | Secondary | 2 (0.9) | 9 (17.3) | 28.12 (5.77 – 136.99) | **<0.001** |
|  | Tertiary | 0 (0) | 4 (7.7) | - | - |
| HH’s education level | No education | 180 (84.9) | 29 (55.8) |  |  |
|  | Primary | 27 (12.7) | 6 (11.5) | 1.38 (0.52 – 3.63) | 0.515 |
|  | Secondary | 5 (2.4) | 12 (23.1) | 14.90 (4.88 – 45.41) | **<0.001** |
|  | Tertiary | 0 (0) | 5 (9.6) | - | - |
| Respondent’s occupation | None | 68 (32.1) | 5 (9.6) |  |  |
|  | Subsistence farmer | 116 (54.7) | 30 (57.7) | 3.52 (1.30 – 9.49) | **0.013** |
|  | Formal | 0 (0) | 7 (13.36) | - | - |
|  | Informal | 11 (5.2) | 8 (15.4) | 9.89 (2.27 – 35.79) | **<0.001** |
|  | Student | 17 (8.0) | 2 (3.9) | 1.6 (0.285 – 8.96) | 0.593 |
| Respondent’s employment | No | 129 (60.8) | 29 (55.8) |  |  |
|  | Yes | 30 (14.15) | 20 (38.46) | 2.97 (1.48 – 5.94) | **0.002** |
|  | Not applicable^ɣ^ | 53 (24) | 3 (5.8) | 0.25 (0.07 – 0.86) | **0.028** |
| HH’s occupation | None | 19 (9.0) | 1 (1.9) |  |  |
|  | Subsistence farmer | 175 (82.6) | 31 (59.6) | 3.37 (0.43 – 26.06) | 0.245 |
|  | Formal | 3 (1.4) | 10 (19.2) | 63.33 (5.81 - 690. 63) | **0.001** |
|  | Informal | 15 (7.1) | 10 (19.2) | 12.67 (1.45 – 110.30) | **0.021** |
| HH employment | No | 167 (78.8) | 28 (53.9) |  |  |
|  | Yes | 43 (20.3) | 24 (46.2) | 3.33 (1.76 – 6.31) | **<0.001** |
|  | Not applicable^ɣ^ | 2 (0.9) | 0 (0) | - | - |
| Marital status^ɸ^ | Single | 23 (10.9) | 10 (19.2) |  |  |
|  | Married | 105 (49.5) | 26 (50) | 0.57 (0.24 – 1.34) | 0.198 |
|  | Divorced/ separated | 10 (4.7) | 5 (9.6) | 1.15 (0.31 – 4.24) | 0.834 |
|  | Widowed | 16 (7.6) | 5 (9.6) | 0.60 (0.21 – 2.51) | 0.604 |
| Meals per day | One | 78 (36.8) | 12 (23.1) |  |  |
|  | Two | 113 (53.3) | 24 (46.2) | 1.38 (0.65 – 2.92) | 0.604 |
|  | Three | 21 (9.9) | 16 (30.1) | 4.95 (2.03 – 12.06) | **<0.001** |
| Meal satisfaction | No | 98 (46.2) | 14 (26.9) |  |  |
|  | Yes | 114 (53.7) | 38 (73.1) | 2.33 (1.19 – 4.56) | **0.013** |
| Alcohol consumption | No | 110 (51.9) | 24 (46.2) |  |  |
|  | Yes | 102 (48.1) | 28 (53.9) | 1.2 (0.68 – 2.31) | 0.459 |

| ɣ | Not applicable was defined as participants under the age of 15 who were not in school or employed |
| --- | --- |
| ɸ | Marital status was not evaluated for participants under 15 who were not married, divorced/separated or widowed |
| HH | Household head |
