# Supplementary material for: Impact of socio-economic factors on Tuberculosis treatment outcomes in north-eastern Uganda: a mixed methods study
Source: BMC Public Health. 2021 Nov 26;21:2167. doi: 10.1186/s12889-021-12056-1 (PMC8620143; doi:10.1186/s12889-021-12056-1)
Supplement: Supplementary file 3 — Additional file 3. Healthcare system factors and TB treatment outcomes. Table of results. [file 12889_2021_12056_MOESM3_ESM.docx]

**Additional file 3:** **Healthcare system factors and TB treatment outcomes**

| **Variable** | | **Unsuccessful treatment N (%)** | **Successful treatment N (%)** | **O.R (95% CI)** | **p-value** |
| --- | --- | --- | --- | --- | --- |
| Distance (km) | <5 | 76 (48.7) | 49 (45.4) | 1 |  |
|  | 5-9 | 36 (23.1) | 21 (19.4) | 1.11 (0.58 – 2.11) | 0.762 |
|  | 10-19 | 20 (12.8) | 21 (19.4) | 0.61 (0.30 – 1.25) | 0.178 |
|  | >=20 | 24 (15.4) | 17 (15.7) | 0.91 (0.44 – 1.87) | 0.797 |
| Transport means | Foot | 138 (88.5) | 92 (85.2) | 1 |  |
|  | Bicycle | 10 (6.4) | 5 (4.6) | 1.33 (0.44 – 4.03) | 0.610 |
|  | Motorcycle/taxi | 8 (5.1) | 11 (10.2) | 0.48 (0.19 – 1.25) | 0.135 |
| Self-rated distance | Short | 33 (21.2) | 31 (28.7) | 1 |  |
|  | Fair | 43 (27.6) | 20 (18.5) | 2.02 (0.98 – 4.16) | 0.056 |
|  | Long | 80 (51.3) | 57 (52.8) | 1.32 (0.73 – 2.39) | 0.364 |
| Healthcare satisfaction | No | 5 (3.2) | 4 (3.7) | 1 |  |
|  | Yes | 151 (96.8) | 104 (96.3) | 1.16 (0.30 – 4.43) | 0.826 |
